# Performance of a Digital Cognitive Assessment in Predicting Dementia Stages Delineated by the Dementia Severity Rating Scale: Retrospective Study

**DOI:** 10.2196/65292

**Published:** 2025-02-26

**Authors:** Duong Huynh, Kevin Sun, Mary Patterson, Reza Hosseini Ghomi, Bin Huang

**Affiliations:** 1 BrainCheck, Inc, Austin, TX, United States; 2 Frontier Psychiatry, PLLC, Billings, MT, United States; 3 Department of Neurology, Institute for Neuroengineering, & eScience Institute, University of Washington, Seattle, WA, United States

**Keywords:** stage, severity, progression, correlation, association, cognitive impairment, functional activities, cognitive assessment, BrainCheck, dementia, Alzheimer disease, gerontology, geriatric, old, elderly, aging, retrospective analysis, digital assessment, patient assessment, digital cognitive assessment, digital health, neurodegeneration, memory loss, memory function, risk factors

## Abstract

**Background:**

Dementia is characterized by impairments in an individual’s cognitive and functional abilities. Digital cognitive assessments have been shown to be effective in detecting mild cognitive impairment and dementia, but whether they can stage the disease remains to be studied.

**Objective:**

In this study, we examined (1) the correlation between scores obtained from BrainCheck standard battery of cognitive assessments (BC-Assess), a digital cognitive assessment, and scores obtained from the Dementia Severity Rating Scale (DSRS), and (2) the accuracy of using the BC-Assess score to predict dementia stage delineated by the DSRS score. We also explored whether BC-Assess can be combined with information from the Katz Index of Independence in activities of daily living (ADL) to obtain enhanced accuracy.

**Methods:**

Retrospective analysis was performed on a BrainCheck dataset containing 1751 patients with dementia with different cognitive and functional assessments completed for cognitive care planning, including the DSRS, the ADL, and the BC-Assess. The patients were staged according to their DSRS total score (DSRS-TS): 982 mild (DSRS-TS 10‐18), 656 moderate (DSRS-TS 19-26), and 113 severe (DSRS-TS 37-54) patients. Pearson correlation was used to assess the associations between BC-Assess overall score (BC-OS), ADL total score (ADL-TS), and DSRS-TS. Logistic regression was used to evaluate the possibility of using patients’ BC-OS and ADL-TS to predict their stage.

**Results:**

We found moderate Pearson correlations between DSRS-TS and BC-OS (*r*=−0.53), between DSRS-TS and ADL-TS (*r*=−0.55), and a weak correlation between BC-OS and ADL-TS (*r*=0.37). Both BC-OS and ADL-TS significantly decreased with increasing severity. BC-OS demonstrated to be a good predictor of dementia stages, with an area under the receiver operating characteristic curve (ROC-AUC) of classification using logistic regression ranging from .733 to .917. When BC-Assess was combined with ADL, higher prediction accuracies were achieved, with an ROC-AUC ranging from 0.786 to 0.961.

**Conclusions:**

Our results suggest that BC-Assess could serve as an effective alternative tool to DSRS for grading dementia severity, particularly in cases where DSRS, or other global assessments, may be challenging to obtain due to logistical and time constraints.

## Introduction

Dementia is characterized by impairments in an individual’s cognitive and everyday functional abilities. Although the pattern and advancement of these impairments vary among patients, the disease is usually considered as having 3 main stages: mild (early-stage), moderate (middle-stage), and severe (late-stage) [1]. The distinction between these categories lies in the extent to which a patient’s physical, cognitive, and psychosocial well-being deteriorates. The rate of deterioration widely varies and progresses through subtle changes in daily functioning to complete loss of independence and the need for a caregiver [1]. Staging of dementia has important implications for practical decision-making and research [2]. From the practical standpoint, knowledge of disease severity is helpful for selection of appropriate intervention options, for prognosis and communication with patients and their family about expectations, care needs, as well as early planning for the future [3-5]. From the research standpoint, staging patients is needed to determine their eligibility for participation, to achieve better clinical efficacy, and to obtain homogeneous sampling in research studies [4], particularly clinical trials.

As noted earlier, dementia is not a monolithic disease; therefore, both cognitive and functional abilities need to be measured to accurately assess its severity and progression. Standardized cognitive tests can provide objective measures of cognitive functioning in different domains such as memory and executive function. Brief cognitive screening tests such as the Montreal Cognitive Assessment and the Mini-Mental State Examination are widely used in clinical practices, but they are rarely used to grade dementia severity [6,7]. Formal neuropsychological tests provide a more comprehensive evaluation of cognitive functioning to support differential diagnoses [8,9]. However, these tests typically take hours and require administration by specialists. Digital cognitive assessments are emerging as an efficient solution due to their self-administration capability, remote accessibility, and automated scoring. Although these types of assessments have been shown to be effective in detecting mild cognitive impairment and dementia [10-12], their ability to stage dementia is not yet clear. Functional assessments quantify the ability to perform activities of daily living through questionnaires such as the Katz Index of Independence in activities of daily living (ADL) [13,14]. Functional assessments are valuable for helping to evaluate dementia severity and also for providing proper guidance to patients and their caregivers.

In clinical practice, both cognitive and functional deficits can be measured using global staging scales. These scales typically come in the form of a questionnaire or interview, relying on subjective judgments and reports from patients or their knowledgeable informants. Compared with other scales, such as the Global Deterioration Scale [15] or the Functional Assessment Staging [16], the Clinical Dementia Rating (CDR) [17-19] scale appears to be the most well-studied and best-evidenced for dementia staging [20,21]. However, the use of this instrument is often impractical in many situations where time and cost are concerns, due to its semi-structured nature, long administration time (30‐60 min), and requirement of clinical judgment from a trained professional during administration and scoring [18,22]. In response to this, brief instruments have been developed to mirror the CDR, including the Dementia Severity Rating Scale (DSRS) [23]. The DSRS uses a multiple-choice format in which the caregiver rates the patient’s cognitive and functional ability in 12 categories [23,24]. The DSRS has been shown to be effective in staging and determining the progression of dementia [23,25,26].

While cognitive tests have been shown to correlate with the above dementia staging tools [4,27,28], previous research primarily focused on traditional paper-based cognitive tests. The increasing adoption of digital solutions and tools in health care calls for the re-evaluation of dementia staging tools and digital cognitive assessments. The first goal of this study was to examine the correlation between scores obtained from the BrainCheck standard battery of cognitive assessments (BC-Assess), a digital cognitive assessment, and scores obtained from the DSRS, a global staging scale. The second goal of this study is to evaluate the effectiveness of using the BC-Assess score to predict dementia stage delineated by the DSRS score. We also explored whether BC-Assess can be combined with information from ADL to obtain enhanced predictive capability.

## Methods

### Data Source

This retrospective study analyzed a real-world dataset of patients who received cognitive care planning services from their providers through BrainCheck Plan. These patients and their caregivers had completed a series of assessments, including DSRS, ADL, and BC-Assess, and received a comprehensive and personalized cognitive care plan. Inclusion criteria for this study were (1) patients 60 years of age or older; (2) assessments of cognitive care planning completed in English on an iPad; and (3) Dementia Severity Rating Scale total score (DSRS-TS) ≥10. The criterion for DSRS-TS was to only include patients that were rated by DSRS to have mild, moderate, and severe dementia [29].

Patients that showed evidence of moderate to severe depression, defined by a Geriatric Depression Scale score of 9 or above [30], were excluded. This is to avoid including reversible causes of dementia, which may have poorly correlated impacts on cognitive and functional measures. Given that depression is common among patients with dementia [31-33], patients with mild depression were not excluded to avoid overfiltering of the data. For patients having multiple care plans, only the latest record was considered. To reduce the impact of practice effect, only data from providers who had completed ≥20 cognitive care planning services for their patients were included. In total, data from 1751 patients with their cognitive care plans completed between February 2022 and April 2024 across 48 providers were included for this analysis.

### Measurements

The DSRS is a brief informant-based questionnaire made up of 12 items that measure functional abilities, including memory, orientation to time, orientation to place, decision-making, social interaction, home activities, personal care, eating, toileting, mobility, recognition, and speech and language. DSRS-TS is calculated by adding up scores across 12 functional areas, ranging from zero (no impairment) to 54 (extreme impairment) [25]. The patients could be categorized into 3 groups of different dementia severity levels based on their DSRS-TS. The majority 56% (n=982) were mild-stage patients (DSRS-TS 10‐18), moderate-stage patients (DSRS-TS 19‐26) accounted for 37.5% (n=656), and the remaining 6.5% (n=113) were severe-stage patients (DSRS-TS 37‐54). The 3 severity levels serve as class labels in a logistic regression analysis in this study to predict patients’ conditions based on their BC-Assess and ADL data.

The BC-Assess, completed by the patients, consisted of 6 individual cognitive assessments: Immediate Recognition and Delayed Recognition (memory), Digit Symbol Substitution (processing speed), Stroop (executive function), Trails Making Test A, and Trails Making Test B (attention or mental flexibility). Detailed descriptions of these tests can be found in previous studies [12,34]. The raw score for each assessment is calculated using assessment-specific measurements based on accuracy or reaction time (Table S1 in Multimedia Appendix 1). The BC-Assess raw overall score is the average of raw scores from all completed assessments after each score has been transformed from its natural range into a common range [0,100] using the formula in Table S1 in Multimedia Appendix 1. A *z*-score is then calculated for each assessment score and for the overall score using the corresponding age- and device-specific mean and standard deviation values from the BrainCheck normative database. Assessment standardized scores and BC-Assess overall standardized score (BC-OS) are reported by rescaling the *z*-scores to have a mean of 100 and a standard deviation of 15.

The ADL is an informant-based 6-item survey that measures an individual’s ability to independently perform basic activities of daily living, including bathing, dressing, going to the toilet, transferring, continence, and feeding. It is calculated by adding up scores from the 6 categories, each of which takes a value of 1 for independent and 0 for dependent, resulting in an ADL total score (ADL-TS) ranging from 0 to 6. An ADL-TS of 2 or less indicates severe functional impairment, 3‐4 indicates moderate impairment, and 5‐6 indicates full function [13,35].

### Statistical Analysis

Data analyses were performed using Python (version 3.8.5). Descriptives were presented for demographics and each score. The *χ*^*2*^ test was used to examine whether the distribution of gender was similar in patients from the 3 groups. The Kruskal-Wallis test was used to compare the mean age of patients across groups.

A 3-way multivariate analysis of variance (MANOVA) was used to examine the joint variation of ADL-TS and BC-OS as a function of dementia stage, age group, and gender. Post-hoc analysis employed 1-way MANOVAs to compare these 2 scores across dementia stages for each individual age and gender group. Tests for statistical significance of the mean differences across stages, age groups, and genders were also performed separately for each score using 3-way ANOVAs. For these statistical comparisons, age is treated as a factor with 3 levels: 60‐69, 70‐79, and 80+.

Logistic regression was used to investigate the effectiveness of using the patients’ BC-OS and ADL-TS to predict their dementia stage, where age and gender served as covariates. In this analysis, age is treated as a continuous variable, and gender is treated as a binary variable: 1 for male and 0 for female. Although the 3 dementia stages form ordinal classes, separate binary logistic regressions were used to classify mild versus moderate; moderate versus severe; and mild versus severe, because the proportional odds assumption was not satisfied for both BC-OS (*P*=.01) and ADL-TS (*P*<.001) from Brant’s Wald test [36], suggesting ordered logistic regression was not appropriate.

The binary logistic regression model for predicting a patient’s condition is:


(1) $$\begin{array}{l} \text{logit}(p)=\beta_{0}+\beta_{1}\cdot st_{BC}+\beta_{2}\cdot st_{ADL}+\beta_{3}\cdot st_{Age}\\ +\beta_{4}\cdot st_{Gender} \end{array}$$

where p is the probability of predicting the patient as having a pre-specified positive class. In each of the above binary classifications, we chose the more severe stage to be the positive class. $st_{BC}$*,* $st_{ADL}$*,* $st_{Age}$*,* $st_{Gender}$ are standardized values of predictor variables BC-OS, ADL-TS, age, and gender. The coefficients $\beta_{i}`s$ (*i*=1‐4) represent the effects of the 4 predictors, and $\beta_{0}$ is the intercept.

Model fitting was based on weighted loss functions to take into consideration class imbalance across the 3 dementia groups. Model performance was evaluated using 5-fold cross-validation with stratification, repeated 20 times (100 iterations), such that on each iteration, all training and testing subsets had roughly the same proportion of patients from each group as in the original dataset. We compared four different models:

(1) full model that included all 4 predictors as in equation (1)

(2) reduced model 1 that included BC-OS and ADL-TS:


(2) $$\text{logit}(p)=\beta_{0}+\beta_{1}\cdot st_{BC}+\beta_{2}\cdot st_{ADL}$$

(3) reduced model 2 that included BC-OS, age, and gender:


(3) $$\text{logit}(p)=\beta_{0}+\beta_{1}\cdot st_{BC}+\beta_{3}\cdot st_{Age}+\beta_{4}\cdot st_{Gender}$$

(4) reduced model 3 that included only BC-OS:


(4) $$\text{logit}(p)=\beta_{0}+\beta_{1}\cdot st_{BC}$$

An receiver operating characteristic (ROC) curve was generated for each model on each cross-validation iteration. Paired-samples *t*-tests were used to compare areas under the receiver operating characteristic curves (ROC-AUC) across models.

### Ethical Considerations

This study was conducted using existing deidentified data collected through BrainCheck. The dataset contained no personal identifiers, and no attempt was made to reidentify the individuals. As such, the research does not meet the definition of “human subjects research” as outlined by HHS 45 CFR 46.102. Therefore, this study did not require ethics review or approval by an institutional review board.

## Results

### Demographics and Assessment Performance

Table 1 summarizes the demographic characteristics of the patients in this study. Group sample size decreased with increasing severity for both genders. The distribution of gender was similar across the 3 groups (*P*=.84). Although the range of age was similar, mean age significantly increased with severity (*P*<.001; pairwise comparisons: *P*<.001 for mild vs moderate and mild vs severe, *P*=.02 for moderate vs severe). Statistical comparisons were not performed for education level and race due to a lot of missing information.

**Table 1.** Demographics and summary statistics of scores across dementia severity groups and the total sample.

Demographic characteristics	Mild group (n=982)	Moderate group (n=656)	Severe group (n=113)	Total (N=1751)
Gender, n (%)^a^				
Female	589 (60)	394 (60.1)	71 (62.8)	1054 (60.2)
Male	393 (40)	262 (39.9)	42 (37.2)	697 (39.8)
Age, years^b^				
Mean (SD)	78 (8.3)	80.3 (8.4)	80.9 (9.0)	79.0 (8.6)
Range	60‐101	60‐102	61‐101	60‐102
Age bucket (years), n (%)^c^				
60‐69	161 (16.4)	74 (11.3)	15 (13.3)	250 (14.3)
70‐79	393 (40)	232 (35.4)	34 (30.1)	659 (37.6)
≥80	428 (43.6)	350 (53.3)	64 (56.6)	842 (48.1)
Education (years), n (%)				
>12	72 (7.3)	49 (7.5)	1 (0.9)	122 (7)
≤12	48 (4.9)	41 (6.3)	9 (8)	98 (5.6)
Not reported	862 (87.8)	566 (86.3)	103 (91.2)	1531 (87.4)
Race, n (%)				
White	108 (11)	72 (11)	6 (5.3)	186 (10.6)
Black	23 (2.4)	12 (1.8)	2 (1.8)	37 (2.1)
Others	19 (1.9)	17 (2.6)	1 (0.8)	37 (2.1)
Not reported	832 (84.7)	555 (84.6)	104 (92.0)	1491 (85.2)
DSRS-TS^d^, mean (SD)	13.4 (2.5)	25.4 (4.8)	43.3 (5.0)	19.8 (9.2)
BC-OS^e^, mean (SD)	62.2 (35.1)	30.1 (38.9)	–5.0 (26.8)	45.8 (41.4)
ADL-TS^f^, mean (SD)	4.8 (1.7)	3.2 (2.1)	1.2 (1.5)	4.0 (2.1)

a *P*=.84 (*χ* test): distribution of gender was not significantly different across groups.

b *P*<.001 (Kruskal-Wallis test): mean age of patients was significantly different across groups.

c *P*<.001 (*χ* test): distribution of age bucket was significantly different across groups.

d DSRS-TS: Dementia Severity Rating Scale total score.

e BC-OS: BrainCheck standard battery of cognitive assessments overall score.

f ADL-TS: activities of daily living total score.

**Figure 1.**
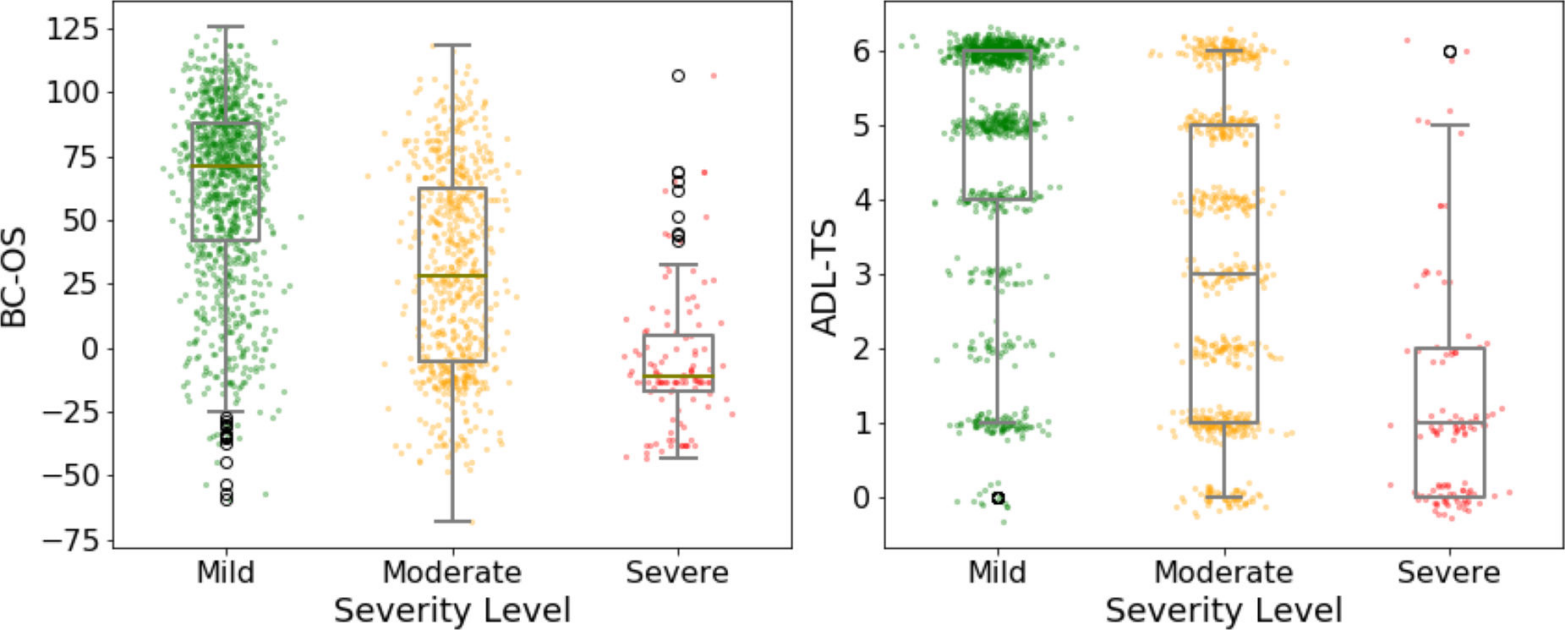
Box plots of the distributions of BrainCheck standard battery of cognitive assessments overall score (BC-OS; left) and activities of daily living total score (ADL-TS; right) for each patient group: green=mild, yellow=moderate*,* and red=severe. Normally distributed random noise was used to add displacements along the x-axis for patients within each group (left and right) and along the y-axis at each ADL-TS value (right).

The means and standard deviations of DSRS-TS, ADL-TS, and BC-OS are provided in Table 1. Overall, the BC-OS and ADL-TS decreased with increasing severity delineated by the DSRS-TS. Figure 1 shows the distributions of BC-OS and ADL-TS across patients within each group. For both scores, the distribution was systematically skewed toward the high values for the mild group, toward the low values for the severe group, and more evenly distributed for the moderate group. Table 2 shows the number and percentage of cases in each group that fall in different BC-Assess and ADL-TS score intervals.

**Table 2.** ADL-TS^a^ and BC-OS^b^ distributions across dementia severity groups.

Score	Mild group (n=982)	Moderate group (n=656)	Severe group (n=113)
BC-OS, n (%)			
Beyond 2 SD of normative mean^c^	480 (48.9)	532 (81.1)	112 (99.1)
Within 2 SD of normative mean	502 (51.1)	124 (18.9)	1 (0.9)
ADL-TS, n (%)			
0‐2 (severe)	137 (14)	275 (41.9)	96 (85)
3‐4 (moderate)	119 (12.1)	142 (21.7)	10 (8.8)
5‐6 (Full function)	726 (73.9)	239 (36.4)	7 (6.2)

a ADL-TS: activities of daily living total score.

b BC-OS: BrainCheck standard battery of cognitive assessments overall score.

c Based on a BC-OS normative mean of 100 and a standard deviation of 15.

### Correlations Between Assessments

We found moderate Pearson correlations between DSRS-TS and BC-OS (*r*=−0.53; *P*<.001), between DSRS-TS and ADL-TS (*r*=−0.55; *P*<.001), and a weak correlation between BC-OS and ADL-TS (*r*=0.37; *P*<.001). Since DSRS covers both cognitive and functional performance of a patient, moderate associations between DSRS-TS with BC-OS and ADL-TS were as expected. The weak correlation between ADL-TS and BC-OS suggests that cognitive and functional abilities are associated with each other, but certain discrepancies exist between the two.

**Figure 2.**
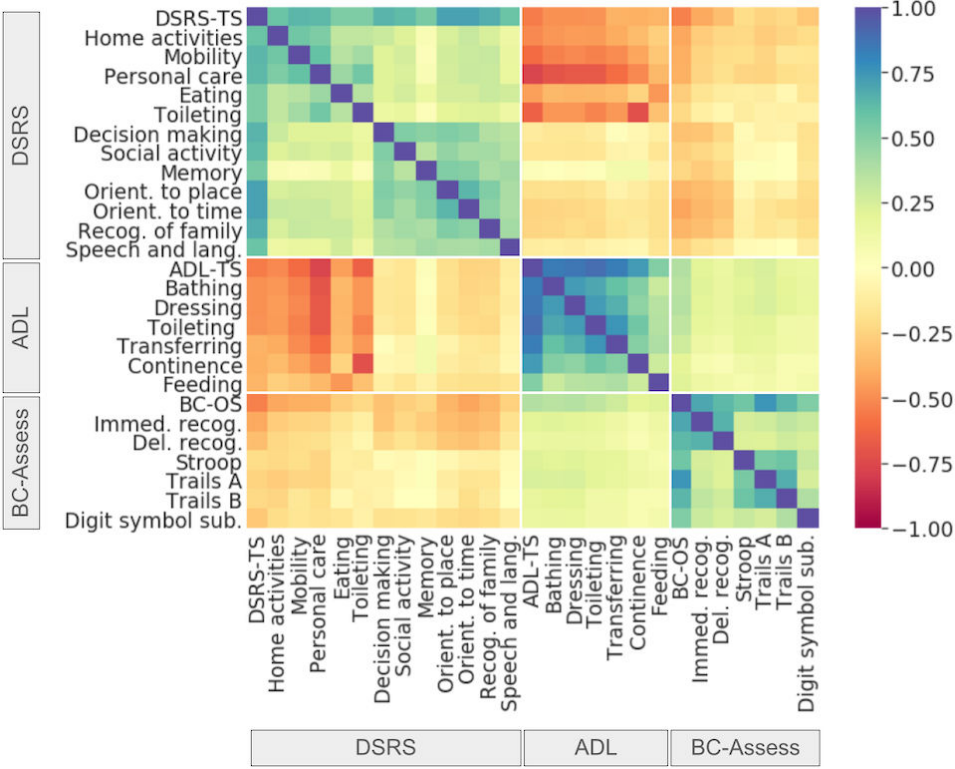
Correlations between BC-Assess (BrainCheck standard battery of cognitive assessments), DSRS (Dementia Severity Rating Scale), and ADL (activities of daily living) subscores. DSRS-TS: DSRS total score; ADL-TS: ADL total score; BC-OS: BC-Assess overall score.

The heatmap in Figure 2 plots Pearson correlations across DSRS, BC-Assess, and ADL subscores. Compared with BC-Assess subscores, BC-OS showed stronger correlations with DSRS and ADL subscores. However, these correlations were weak. Among DSRS subscores, BC-OS was most associated with home-activities (*r*=−0.41; *P*<.001), mobility (*r*=−0.39; *P*<.001), personal-care (*r*=−0.39; *P*<.001), orientation-to-time or orientation-to-place (*r*=−0.43 and −0.37; *P*<.001), and recognition-of-family (*r*=−0.37; *P*<.001). Among ADL subscores, BC-OS was most associated with dressing (*r*=0.36; *P*<.001), bathing (*r*=0.35; *P*<.001), and toileting (*r*=0.33; *P*<.001). Although weaker, a clear trend can be observed from Figure 2 for BC-Assess subscores. With regard to the DSRS, assessments of memory (Immediate or Delayed Recognition) were most associated with memory-demanding activities such as memory, orientation-to-time, orientation-to-place, recognition-of-family, and decision-making, whereas assessments of executive function, attention, or mental flexibility (Stroop, Trails Making A/B) were most associated with home-activities, mobility, and personal-care. With regard to the ADL, BC-Assess subscores were more associated with bathing, dressing, and toileting than with categories that are more essential, such as feeding, continence, and transferring. Between ADL and DSRS subscores, correlations mainly occurred within a subset of DSRS activities that are of the same types as those rated by the ADL such as eating, home-activities, mobility, personal-care, and toileting. Of these, the strongest correlations were found between DSRS toileting and ADL continence (*r*=−0.72; *P*<.001), and between DSRS personal-care and ADL bathing (*r*=−0.70; *P*<.001), dressing (*r*=−0.68; *P*<.001), and toileting (*r*=−0.68; *P*<.001).

### Comparison of BC-OS and ADL-TS Across Dementia Stages, Age Groups, and Genders

Given the correlation between BC-OS and ADL-TS, we analyzed the differences in these two scores across dementia stages, age groups, and genders by running a 3-way MANOVA (BC-OS + ADL-TS~ Dementia Stage * Age Group * Gender). Results based on the Pillais Trace method showed a significant effect of Dementia Stage (Pillais Trace =0.046 , *F*_43,466_=20.5; *P*<.001) whereas the effects of age and gender and all interaction terms were not significant. Post hoc 1-way MANOVAs (*BC-OS + ADL-TS~ Dementia Stage*) were run to compare BC-OS and ADL-TS combined between mild versus moderate and between moderate versus severe separately for each age and gender group. Except for the 60‐69 and female group (n=136) showing a nonsignificant difference between moderate versus severe, significant differences were observed for all other cases. We further performed 3-way ANOVAs where BC-OS (BC-OS~ Dementia Stage * Age Group * Gender) and ADL-TS (ADL-TS~ Dementia Stage * Age Group * Gender) were considered separately. For BC-OS, we found a significant main effect of Dementia Stage only (*F*_21,733_=270.31; *P*<.001). The insignificant differences in BC-OS across age groups were as expected as this score had been standardized to adjust for age differences. For ADL-TS, we found significant effects of Dementia Stage (*F*_21,733_=278.87; *P*<.001), Age Group (*F*_21,733_=4.77; *P*=.009), and Gender (*F*_11,733_=7.82; *P*=.005). For both scores, no interaction terms were significant.

### Logistic Regression to Examine the Roles of BC-OS, ADL-TS, Age, and Gender in Predicting a Patient’s Condition

**Figure 3.**
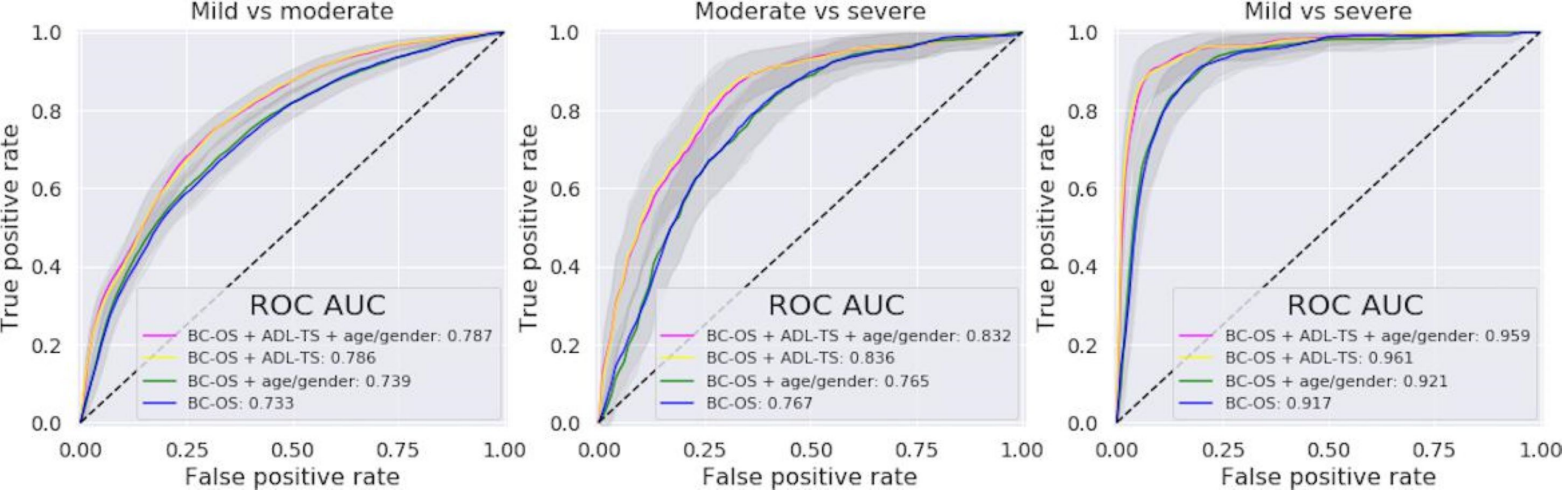
Model comparisons for the classification between mild and moderate (left) and between moderate and severe (right): receiver operating characteristic curves and mean area under the receiver operating characteristic curve (ROC AUC) for the full model (BrainCheck standard battery of cognitive assessments overall score [BC-OS] + activities of daily living total score [ADL-TS] + age and gender; magenta), reduced model 1 (BC-OS + ADL-TS; yellow), reduced model 2 (BC-OS + age and gender; green), and reduced model 3 (BC-OS only; blue). The shaded area along each curve represents the corresponding standard deviation across cross validation iterations. In each classification, the more severe condition was chosen to be the positive class.

ROC analysis (Figure 3) showed a comparable performance between the full model (*BC-OS + ADL-TS + age or gender*: ROC-AUC=0.787 for mild vs moderate; 0.832 for moderate vs severe; and 0.959 for mild vs severe) and reduced model 1 (*BC-OS + ADL-TS*: ROC-AUC=0.786 for mild vs moderate; 0.836 for moderate vs severe; and 0.961 for mild vs severe), and between reduced model 2 (*BC-OS + age or gender*: ROC-AUC=0.739 for mild vs moderate; 0.765 for moderate vs severe; and 0.921 for mild vs severe) and reduced model 3 (*BC-OS* only: ROC-AUC=0.733 for mild vs moderate; 0.767 for moderate vs severe; and 0.917 for mild vs severe). The small differences in ROC-AUC generated by the omission of age and gender suggest that they are not important predictors. Moreover, including these demographic factors appears to have led to some degree of overfitting where reduced model 1 performed slightly but significantly better than the full model (mild vs severe: *P*=.002; moderate vs severe: *P*<.001) and reduced model 3 performed slightly but significantly better than reduced model 2 (mild vs moderate: *P*<.001; moderate vs severe: *P*=.04; mild vs severe: *P*<.001).

The 2 models that include ADL-TS (full model and reduced model 1) performed significantly better than the 2 models without ADL-TS (reduced models 2 and 3) (*P*<.001 for each pairwise comparison), suggesting the important role of ADL. For all binary classifications, although BC-OS alone can serve as a fairly effective predictor with an ROC-AUC of at least 0.733, including ADL-TS in the model significantly improved prediction accuracy.

As age and gender were not important factors, we excluded them from further analysis. We examined the diagnostic performance of reduced models 1 (*BC-OS + ADL-TS*) and 3 (*BC-OS* only) at the optimal cut-points from their ROC curves. These are points on the ROC curves that maximize true positive rate and minimize false positive rate. Table 3 shows sensitivity (true positive rate) and specificity (1–false positive rate) at the optimal cut-point for each model and classification. When included, ADL-TS improved the sensitivity and specificity by 3%‐5%. For reduced model 3, which included only BC-OS, we found that the optimal cut-point corresponded to a BC-OS of 52 (roughly 3 standard deviations below the normative mean) for the classification between mild versus moderate, and a BC-OS of 0 for the classification between moderate versus severe.

**Table 3.** Sensitivity (Se) and specificity (Sp) by model and classification: mean (SD) across cross-validation iterations. In each classification, the more severe condition was chosen to be the positive class.

Classification	BC-OS^a^ + ADL-TS^b^		BC-OS only	
	Se, mean (SD)	Sp, mean (SD)	Se, mean (SD)	Sp, mean (SD)
Mild versus moderate	0.74 (0.04)	0.72 (0.04)	0.69 (0.05)	0.68 (0.05)
Moderate versus severe	0.84 (0.06)	0.76 (0.05)	0.77 (0.07)	0.72 (0.07)
Mild versus severe	0.92 (0.04)	0.93 (0.03)	0.89 (0.04)	0.86 (0.04)

a BC-OS: BrainCheck standard battery of cognitive assessments overall score.

b ADL-TS: activities of daily living total score.

The fitted model coefficients are provided for reduced models 1 and 3 in Table 4. The 1-sided *P* value obtained from bootstrapping for each coefficient is shown in the parentheses.

**Table 4.** Coefficients (*P* values) of the fitted reduced models 1 (*BC-OS^a^* *+ ADL-TS^b^*) and model 3 (*BC-OS* only).

	Regression coefficients, β (*P* value)				
	Reduced model 1			Reduced model 3	
Classification	$\beta_{0}$ (Intercept)	$\beta_{1}$ (${st}_{BC}$)	$\beta_{2}$(${st}_{ADL}$)	$\beta_{0}$ (Intercept)	$\beta_{1}$ (${st}_{BC}$)
Mild versus moderate	−0.071 (.01)	−0.757 (<.001)	−0.717 (<.001)	−0.053 (.002)	−0.890 (<.001)
Moderate versus severe	−0.819 (<.001)	−0.989 (.03)	−1.059 (<.001)	−0.453 (<.001)	−1.215 (<.001)
Mild versus severe	−2.443 (<.001)	−1.611 (<.001)	−1.517 (<.001)	−1.557 (<.001)	−2.076 (<.001)

a BC-OS: BrainCheck standard battery of cognitive assessments overall score.

b ADL-TS: activities of daily living total score.

## Discussion

By conducting a retrospective analysis of patient data in real-world clinical settings, this study sought to investigate the relationship between cognitive performance in a battery of digital cognitive assessments, BC-Assess, and dementia severity measured by the DSRS. We found a statistically significant moderate correlation between the BC-OS and the DSRS-TS. This correlation is comparable with that between the ADL-TS and the DSRS-TS. Both BC-OS and ADL-TS significantly decrease with increasing severity. BC-Assess demonstrated to be a good predictor of dementia severity, with ROC-AUC of classification using logistic regression ranging from 0.733 to 0.917. When BC-Assess was combined with ADL, higher prediction accuracies were achieved, with ROC-AUC ranging from 0.786 to 0.961.

Our results suggest that BC-Assess could serve as an alternative tool to DSRS for grading dementia severity, particularly in cases where DSRS, or other global assessments, may be challenging to obtain due to logistical and time constraints. Unlike DSRS, BC-Assess, as a brief digital cognitive assessment, offers the advantage of flexible choice of self-administration or administration by clinical support staff, runs on common consumer technology, and does not require availability of an informant. The significant improvement of prediction accuracies when BC-Assess is combined with ADL indicates the synergetic relationship between cognitive and functional measures in grading dementia severity. Previous studies have shown that patients’ loss of independence to manage activities of daily living is nonlinearly related with their cognitive decline [37], and their correlation weakens as the disease progresses [38]. This is consistent with the finding of relatively low correlation (*r*=0.37) between the 2 measures in this study and elsewhere [39,40]. Together, these findings suggest that ADL carries additional information of functional abilities that is partially independent of cognitive abilities measured by BC-Assess. When combined, the 2 measures provide a more comprehensive understanding of a patient’s condition.

While the BC-OS and the ADL-TS from the mild and severe groups separate well from each other, scores from the moderate group vary widely among patients and largely overlap with both the mild and severe groups. This is reflected in high sensitivity and specificity (.86 or higher) for the classification between the mild and severe groups, and moderate sensitivity and specificity (0.83 or lower) for the classifications of the moderate group. Overall, however, a gradual change in the distribution of each score across stages can be observed. In line with current knowledge about the progression and stages of dementia [1,41], this pattern of results suggests that cognitive and functional declines do not happen in the same way across patients with dementia, and that there might only be subtle differences in cognitive or functional performance, or both, across patients within 2 successive stages.

Implicit in this study is the assumption that the staging of dementia severity based on the DSRS-TS had accurately identified each patient’s underlying degree of severity. Previous studies demonstrated that the DSRS has high test-retest and inter-rater reliability [25] and good concurrent validity with high correlations with the CDR, the Mini-Mental Status Examination, and the Consortium to Establish a Registry for Alzheimer’s Disease [23,25]. Other studies showed that the DSRS-TS can effectively differentiate between individuals with dementia, MCI, and healthy controls [42], and that it changes at a constant linear rate throughout the entire clinical course of dementia [25]. However, the psychometric properties of the DSRS in distinguishing between patients with mild, moderate, and severe dementia have not been studied. The DSRS-TS cut-offs used for staging of dementia severity have been suggested based on the mapping of the DSRS-TS onto CDR stages where a CDR global score of 0, 0.5, 1, 2, and 3 represent no, questionable, mild, moderate, and severe dementia. However, it has been shown that there is a large variability in the degree of severity among patients placed in a particular CDR stage, and patients with the same degree of severity can be placed in different CDR stages [2,43,44]. Furthermore, the precision of severity grading depends on the scoring approach to the CDR, ie, the item-response-theory approach is more precise than the sum-of-the-boxes approach, which is more precise than the global score approach [43]. On top of that, the mapping of DSRS-TS to CDR global score was based on a limited sample of patients with dementia that might not be representative of patients at different severity stages in general [23]. These limitations are possible contributing factors to the widespread distributions and overlaps of BC-OS and ADL-TS across patient groups delineated by the DSRS-TS in this study. To allow for more systematic investigations into the effectiveness of using these scores in dementia staging, future research needs to address these limitations and establish more fine-grained and well-defined staging criteria as well as optimize the scoring methods for the DSRS, the CDR, and other assessments of dementia severity.

Suboptimal and inconsistent data quality in a dataset acquired outside typical clinical research settings is another factor that potentially causes large variabilities of scores observed in this study [45]. While real-world data may better represent diverse clinical environments, which is desired to obtain a generalized relationship between assessments, limited control over the data collection process and differences in clinical practices may result in reduced accuracy and consistency of the data. Inconsistencies exist across clinical sites and across units within each site due to differences in internal policies, staff training, workflow, and expertise. Inconsistencies also come from the different patient or caregiver populations each site serves. Patients may also have different dementia pathologies, leading to significant differences in the pattern of scores.

The ADL and BC-Assess also have their own limitations that one should take into consideration when interpreting the current results. The ADL measures 6 basic activities of daily living and employs dichotomous scoring, which allows only 2 possible scores for each functional category, ie, 1 for *independent* and 0 for *dependent*. Therefore, it lacks the resolution to capture intermediate levels of dependence. Furthermore, as it is subjective ratings from informants, for cases with small changes in functional activities, patients may end up receiving substantially different scores depending on their caregivers’ judgments, resulting in low interrater variability [46,47]. In this study, with only patients with dementia included (based on their DSRS-TS), we found high variability in the ADL-TS across patients within each group, especially for the moderate group. As for the BC-Assess, besides measurement errors that exist in any assessment and technical difficulties older adults may have when using smart devices for the assessment, it might have limited utility in severity staging because patients with extremely severe conditions might not be able to complete it [48], and it could also suffer from a floor effect, a common limitation of psychometric tests [49].

Our data show a high imbalance in the number of patients across the 3 groups, with mild, moderate, and severe dementia accounting for 56%, 37.5%, and 6.5%, respectively. Although the trend is similar, higher proportions of patients estimated to be in the moderate (31%) and severe (21%) stages of Alzheimer’s Disease were reported in a previous study [50]. As patients included in this study were those that received cognitive care planning services from their providers through the BrainCheck Plan platform within a 2-year period, our data do not necessarily reflect the prevalence of each stage. The fact that we only included patients with DSRS, ADL, and BC-Assess data also limited the number of patients in later stages who might be too impaired to take the BC-Assess. Furthermore, given its main goals are to help patients and their families better understand the patients’ condition and needs, to offer strategies to improve their overall quality of life, and to plan for the future when their condition gets worse, cognitive care planning is more meaningful for patients in early stages. Patients in the severe stage of dementia are completely dependent on their family or caregivers, and many of them require specialized care and attention in facilities. These institutionalized patients tend to have been diagnosed and given care plans tailored to their specific needs by the institution.

In addition to the findings of this study, the growing field of ecological digital assessment tools offers valuable insights into monitoring and predicting dementia progression using digital biomarkers in real-world settings. For example, Buegler et al [51] show how these tools provide individualized, context-sensitive data to better understand cognitive performance[Bibr R49]. Integrating ecological tools with measures like BC-Assess could enhance its utility by capturing real-time data and offering a more comprehensive view of a patient’s condition. These tools could also address challenges in tracking cognitive and functional abilities over time, particularly when in-person assessments or informants are unavailable. Further research into combining ecological tools with BC-Assess could refine dementia severity assessments and improve patient outcomes across stages of the disease.

Despite the limitations, this study shows that BC-Assess could be a promising solution for measuring dementia severity. The use of BC-Assess for this purpose will be particularly useful in primary care settings, where DSRS or other comprehensive global assessments may pose implementation challenges. Due to its flexibility, efficiency, and ease of use, BC-Assess can help streamline the assessment process, supporting timely diagnosis and management of dementia. This, in turn, can improve patient outcomes and ease the burden on caregivers.

## Supplementary material

10.2196/65292 Multimedia Appendix 1 Raw score (RS) metric and transformed score (TS) calculation for each assessment.

## Abbreviations

ADL: activities of daily living
ADL-TS: activities of daily living total score
BC-Assess: BrainCheck standard battery of cognitive assessments
BC-OS: BrainCheck standard battery of cognitive assessments overall score
CDR: Clinical Dementia Rating
DSRS: Dementia Severity Rating Scale
DSRS-TS: Dementia Severity Rating Scale total score
MANOVA: multivariate analysis of variance
ROC: receiver operating characteristic
ROC-AUC: area under the receiver operating characteristic curve
