# Supplementary material for: Performance of a Digital Cognitive Assessment in Predicting Dementia Stages Delineated by the Dementia Severity Rating Scale: Retrospective Study
Source: JMIR Aging. 2025 Feb 26;8:e65292. doi: 10.2196/65292 (PMC11882104; doi:10.2196/65292)
Supplement: Multimedia Appendix 1 [file aging-v8-e65292-s001.docx]

# Supplementary Information

Table S1. Raw score (RS) metric and transformed score (TS) calculation for each assessment.

| **Assessment** | **Raw Score (RS) Metric** | **Transformed Score (TS)** |
| --- | --- | --- |
| Immediate/Delayed  Recognition | Number of correct  responses | TS = 100*RS/MAX^a^ |
| Trails Making A | Median reaction time | TS = 100*(1-RS/MAX) |
| Stroop | Median reaction time | TS = 100*(1-RS/MAX) |
| Digit Symbol  Substitution | Number of correct  responses per second | TS = 100*RS/MAX |

^a^MAX represents the population maximum score of the assessment across all individuals in the BrainCheck normative database.
